# A Steep-Slope Transistor Combining Phase-Change and Band-to-Band-Tunneling to Achieve a sub-Unity Body Factor

**DOI:** 10.1038/s41598-017-00359-6

**Published:** 2017-03-23

**Authors:** Wolfgang A. Vitale, Emanuele A. Casu, Arnab Biswas, Teodor Rosca, Cem Alper, Anna Krammer, Gia V. Luong, Qing-T. Zhao, Siegfried Mantl, Andreas Schüler, A. M. Ionescu

**Affiliations:** 1 Nanoelectronic Devices Laboratory (NanoLab), École Polytechnique Fédérale de Lausanne (EPFL), 1015 Lausanne, Switzerland; 2 Solar Energy and Building Physics Laboratory (LESO-PB), École Polytechnique Fédérale de Lausanne (EPFL), 1015 Lausanne, Switzerland; 3 Peter Grünberg Institut 9 (PGI-9), Forschungszentrum Jülich, 52425 Jülich, Germany

## Abstract

Steep-slope transistors allow to scale down the supply voltage and the energy per computed bit of information as compared to conventional field-effect transistors (FETs), due to their sub-60 mV/decade subthreshold swing at room temperature. Currently pursued approaches to achieve such a subthermionic subthreshold swing consist in alternative carrier injection mechanisms, like quantum mechanical band-to-band tunneling (BTBT) in Tunnel FETs or abrupt phase-change in metal-insulator transition (MIT) devices. The strengths of the BTBT and MIT have been combined in a hybrid device architecture called phase-change tunnel FET (PC-TFET), in which the abrupt MIT in vanadium dioxide (VO_2_) lowers the subthreshold swing of strained-silicon nanowire TFETs. In this work, we demonstrate that the principle underlying the low swing in the PC-TFET relates to a sub-unity body factor achieved by an internal differential gate voltage amplification. We study the effect of temperature on the switching ratio and the swing of the PC-TFET, reporting values as low as 4.0 mV/decade at 25 °C, 7.8 mV/decade at 45 °C. We discuss how the unique characteristics of the PC-TFET open new perspectives, beyond FETs and other steep-slope transistors, for low power electronics, analog circuits and neuromorphic computing.

## Introduction

Complementary metal-oxide semiconductor (CMOS) technology has been the core of micro/nanoelectronics industry for decades. In the Dennardian scaling era of MOS transistors, extraordinary improvements in terms of switching speed, device density, functionality and cost have been achieved by the additive application of several technology boosters such as substrate engineering, strain, multi-gate, high-k/metal gate stacks and high-mobility channel materials. However, the concept of a metal-oxide-semiconductor field-effect transistor (MOSFET) remained unchanged. Recently, aggressive scaling of the gate length dimensions down to few tens of nanometers is facing major challenges in terms of process variability, high leakage power, unscalable voltage supply and degraded current switching ratios^1^.

The quest for a new beyond CMOS switch, addressing essentially leakage power and voltage scaling, encompasses new device concepts and materials, capable to complement MOSFETs and to be integrated on advanced CMOS platforms^2, 3^. A fundamental target is the reduction of the subthreshold swing *SS* (=*dV*_g_/*d*log*I*_d_), which in a conventional MOSFET is limited to 60 mV/decade at room temperature (*T* = 300 K) due to the thermionic carrier injection mechanism^4^. A steep-slope switch, with *SS* < 60 mV/decade, would allow to scale down the supply voltage and to enable future low-power computing^5^. Different steep-slope device principles have been proposed for this purpose, exploiting negative capacitance^6^, movable electro-mechanical gates^7^, impact ionization^8^ and tunnel field-effect transistors (TFETs) based on quantum mechanical band-to-band tunnelling^9^ (BTBT). TFET is currently considered the most promising steep-slope solid-state switch among alternative technologies, with experimentally demonstrated *SS* values of the order of 30 mV/decade at room temperature^10^ mainly limited into a range of low currents. However, the tunnelling conduction mechanism limits the device performance in terms of ‘on’ current, *I*_ON_, and the frequency of operation.

Recently, phase change materials such as correlated functional oxides have been proposed as a promising solution for beyond CMOS electronics. External excitations applied to phase change materials can induce a phase transition accompanied by a drastic change in their conduction properties^11–19^. One of the most studied phase change materials is vanadium dioxide (VO_2_), which exhibits a metal-insulator transition (MIT) corresponding to a structural phase transition at a critical temperature *T*_MIT_ (340 K in bulk VO_2_^20–22^). When VO_2_ temperature is increased above *T*_MIT_, the material transitions from a monoclinic phase to a tetragonal rutile structure, concomitant with the closing of an energy gap *E*_g_ ≈ 0.6 eV in the 3d conduction band and a steep decrease in resistivity, up to 5 orders of magnitude in bulk VO_2_. When the VO_2_ temperature is decreased, the transition back to the monoclinic phase is observed for values below *T*_MIT_, giving rise to a hysteresis with width depending on the quality of the material. VO_2_ holds great potential for beyond CMOS electronics because the MIT can be induced by electrical excitations, enabling applications based on volatile resistive switching. The VO_2_-based MIT switch in 2-terminal configuration shows interesting properties such as abrupt increase in current with applied voltage^23–31^, fast switching time^32–34^, high reliability^35, 36^, negative differential resistance^37–40^, memristive switching^41, 42^ and low temperature dependence of transition dynamics^43, 44^. However, the main drawback of the 2-terminal MIT switch is the relatively high leakage current *I*_OFF_ due to the small bandgap of VO_2_ in the insulating state. While this problem can be mitigated by VO_2_ doping^45^, the most effective solution would be the development of 3-terminal switches in which a VO_2_ channel undertakes a gate-driven phase change. The development of such a device was attempted first with standard MOSFET structures using VO_2_ as the semiconducting material^46^, but the observed conductance modulation by gate voltage was limited to a small percentage^47–50^. This encouraged the investigation of the use of electrolyte gating to obtain very high electric fields at the interface between VO_2_ and an ionic liquid^51, 52^, inducing a higher channel conductance modulation due to the creation of oxygen vacancies^53–55^ or protonation^56^ but with a much slower switching time^57, 58^.

**Figure 1:**
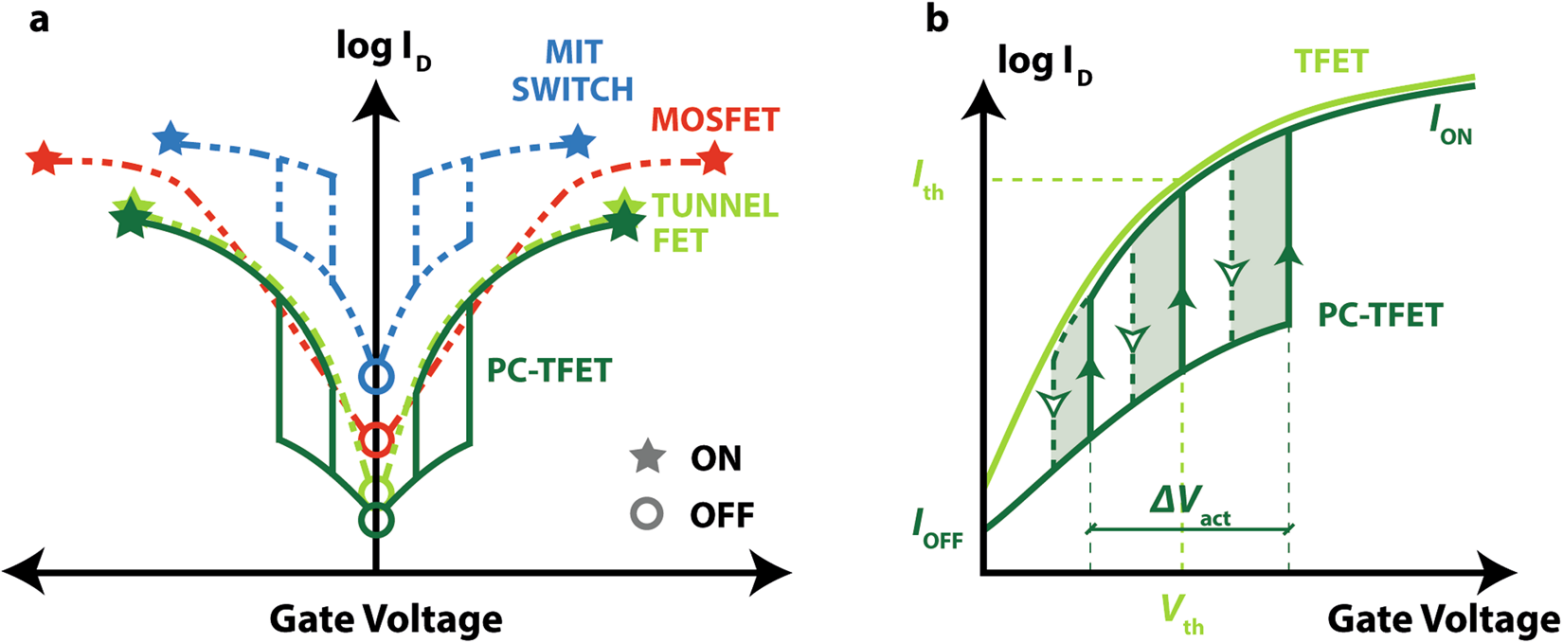
Comparison of three steep-slope switches: TFET, MIT and proposed PC-TFET. (**a**) Transfer characteristics for the PC-TFET and other steep-slope switches (TFET, MIT switch) achieving lower subthreshold swing than the MOSFET. (**b**) PC-TFET characteristics for different values of the MIT switch threshold voltage *V*_act_, compared to the transfer characteristics of the TFET component with threshold voltage *V*_th_. The hysteresis areas for three different values of *V*_act_ are highlighted by the shaded regions.

In order to overcome these issues, the phase-change tunnel FET (PC-TFET) has been proposed^59^ as a hybrid design integration of a tunnel FET and a 2-terminal MIT switch, combining the strengths of the two devices and resulting in the first solid-state VO_2_-based 3-terminal switch with simultaneous very low *I*_OFF_ current, high *I*_ON_/*I*_OFF_ ratio and ultra-steep subthreshold swing (Fig. 1a), performance that cannot be individually achieved by a TFET or a MIT switch. The transfer characteristics of the PC-TFET are qualitatively compared to the ones of the TFET used as a component part in Fig. 1b. The main working principle of the PC-TFET is to feedback (by an appropriate gate or source connection) the ultra-abrupt switching in the MIT material into a TFET characteristic, used to block the current in the OFF state. The phase change in the MIT switch corresponds to the actuation voltage *V*_act_ (tunable by the design of the MIT component) allowing to switch from a high resistance state to a low resistance state, in which the current follows the transfer characteristics of the TFET. For ideal performance, the *V*_act_ of a 2-terminal MIT switch should be aligned with the TFET threshold voltage *V*_th_ (defined by the constant current method). Figure 1a and b also depict the resulting hysteretic behaviour of the PC-TFET, inherited from its MIT component. In this work, we discuss in detail the PC-TFET principle, its integration and the method of extraction of the body factor. Moreover, we further characterize the PC-TFET to discuss its temperature dependence and possible applications for analog circuits and neuromorphic computing.

## Results

### Hybrid PC-TFET: principle

The principle of the PC-TFET steep slope hybrid device is to simultaneously use two physical mechanisms to lower the subthreshold swing factors *m* and *n*, respectively the body factor (mirroring the differential amplification of surface potential) and the carrier injection mechanism in the conduction channel (by band-to-band-tunnelling in a gated p-i-n junction):

1 $$SS=\frac{{\rm{d}}{V}_{{\rm{GS}}}}{{\rm{d}}({\mathrm{log}}_{10}{I}_{{\rm{DS}}})}=\mathop{\overbrace{\frac{{\rm{d}}{V}_{{\rm{GS}}}}{{\rm{d}}{V}_{\mathrm{GS}\_\mathrm{INT}}}\frac{{\rm{d}}{V}_{\mathrm{GS}\_\mathrm{INT}}}{{\rm{d}}{{\Psi }}_{{\rm{S}}}}}}\limits^{m}\mathop{\overbrace{\frac{{\rm{d}}{{\Psi }}_{{\rm{S}}}}{{\rm{d}}({\mathrm{log}}_{10}{I}_{{\rm{DS}}})}}}\limits^{n}$$

while the use of band-to-band tunnelling is intrinsically offering a straightforward solution to a potentially lower than 60 mV/decade *n*-factor, for lowering *m*, in contrast with any other previous reports, we do not use any negative capacitance principle but a simple circuit technique exploiting the abrupt switching in a 2-terminal MIT device connected in a voltage divider placed in the gate or in the source of a TFET. It is worth noting that reducing the body factor, *m*, of a TFET below 1, corresponds to a less explored approach (previously proposed by Ionescu^60^) to boost the abruptness of subthreshold characteristics of a TFET.

**Figure 2:**
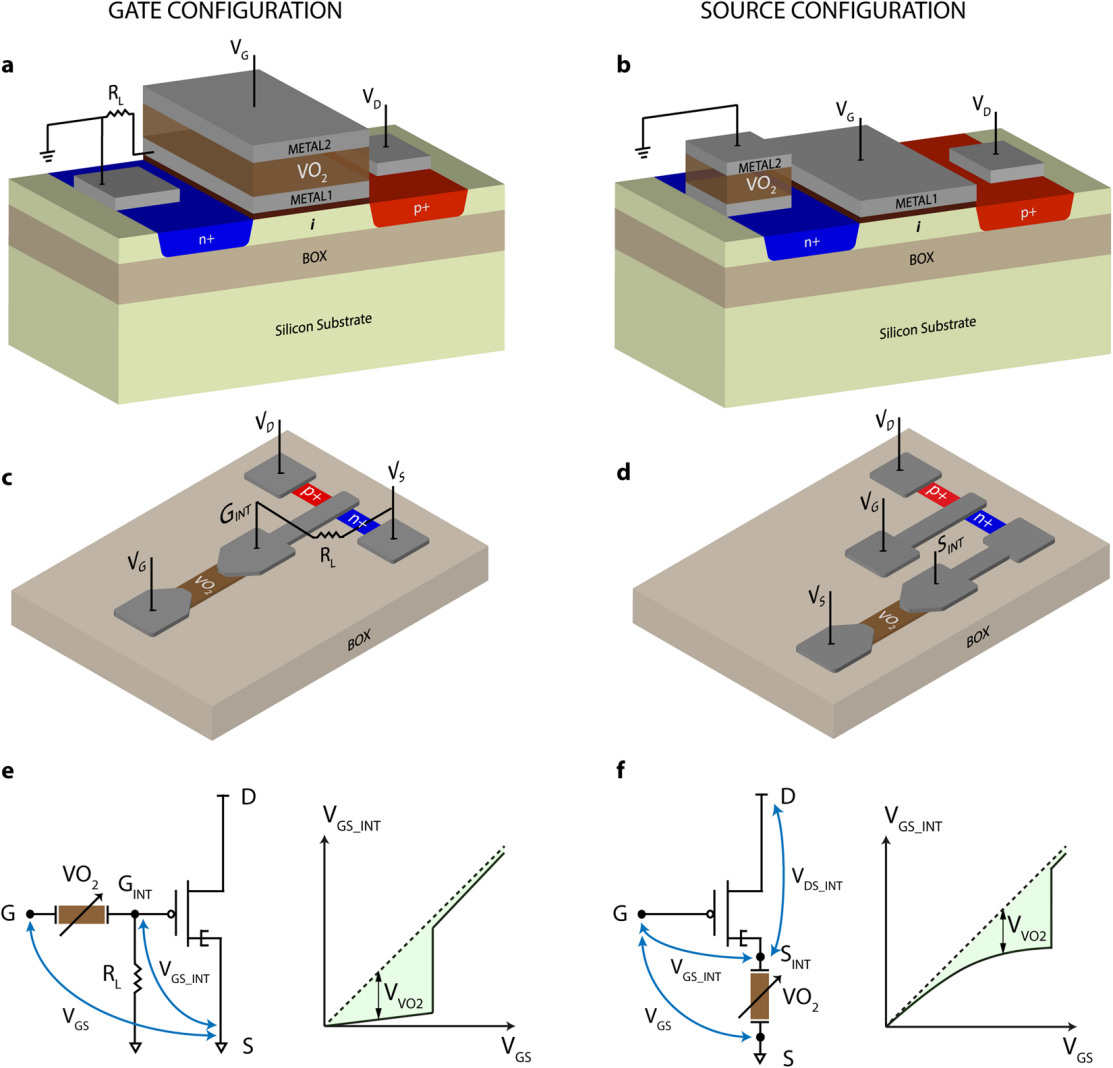
Phase change TFET integration in gate and source configuration. (**a**,**b**) 3D schematic diagrams of the PC-TFET integrating vertical VO_2_ switches. (**c**,**d**) 3D schematic diagrams of the PC-TFET integrating planar VO_2_ switches. (**e**,**f**) Equivalent circuits showing the internal TFET gate voltage *V*_GS_INT_ amplified by the MIT switch phase change, induced by the external gate voltage *V*_GS_.

In the following, we study two PC-TFET designs, in which the MIT switch is connected to the gate (Fig. 2a,c,e, “gate configuration”) or to the source (Fig. 2b,d,f, “source configuration”) terminal of the TFET. In both cases the state of the MIT switch is controlled by the gate voltage *V*_GS_ and the phase change induces an internal differential amplification of the voltage drop *V*_GS_INT_ between the gate and source terminals of the TFET (d*V*_GS_INT_/*V*_GS_ >> 1) resulting in a steep increase in current *I*_DS_.

Figure 2a shows the hybrid design integration of a 3-terminal TFET and a 2-terminal VO_2_ switch to obtain the PC-TFET gate configuration. A VO_2_ thin film is deposited and patterned on top of the gate terminal of the TFET, and a second metal layer is used to contact it and define the gate electrode of the PC-TFET. The same design can be adapted to the source configuration, shown in Fig. 2b, where the VO_2_ switch is built on top of the source terminal of the TFET. An alternative design exploiting planar VO_2_ switches is reported in Fig. 2c for the gate configuration and Fig. 2d for the source configuration.

Figure 2e presents the equivalent circuit and voltage distribution for the PC-TFET in gate configuration. A load resistance *R*_L_ is used to allow a current flow high enough to reach the power threshold of the VO_2_ switch^43^. The value of *R*_L_ is selected in order to have *R*_VO2_OFF_ >> *R*_L_ >> *R*_VO2_ON_, where *R*_VO2_OFF_ is the resistance of the MIT switch in the insulating state and *R*_VO2_ON_ is the resistance in the metallic state. As *V*_GS_ is ramped up in this configuration, the VO_2_ material is initially in the highly resistive state, hence most of the voltage drops on the MIT switch (*V*_VO2_ ≈ *V*_GS_) and *V*_GS_INT_ stays low. Once the voltage is high enough to induce the metallic state in VO_2_, *V*_VO2_ drops to a very low value and *V*_GS_INT_ experiences a steep transition to a value approaching *V*_GS_.

Figure 2f presents the equivalent circuit and voltage distribution for the PC-TFET in source configuration. In this case the MIT switch is connected to the internal source terminal of the TFET and both the internal voltage drops *V*_GS_INT_ and *V*_DS_INT_ are changing while sweeping *V*_GS_ depending on *V*_VO2_, such that *V*_GS_ − *V*_GS_INT_ = *V*_DS_ − *V*_DS_INT_ = *V*_VO2_. For low values of *V*_GS_, the VO_2_ material is in its insulating state but the TFET channel resistance is much higher, effectively blocking the leakage through the MIT switch and keeping a low *I*_OFF_ current. Hence *V*_GS_INT_ follows *V*_GS_. Increasing *V*_GS_, the tunnelling current increases steadily until the TFET resistance becomes comparable with *R*_VO2_OFF_. At this point the rise in *V*_GS_INT_ decreases and the MIT switch approaches its power threshold. Once VO_2_ switches to its metallic state, *V*_GS_INT_ jumps abruptly to values near *V*_GS_. It is clear that the source configuration is very suitable for the lowest power consumption and aggressive scaling as it does not require any additional load resistor (which is the TFET itself) and there is no power dissipation in such a load. However, as it will be shown later, the gate configuration is particularly interesting for its steeper characteristics.

The source configuration is similar to a previously reported solution based on III–V FinFET transistors and VO_2_ switches^61^. However, that work exploited classical FinFETs with thermionic subthreshold swing and with very high leakage current to induce the phase change in VO_2_, and as a consequence the *I*_ON_/*I*_OFF_ ratio was limited to 4 × 10^2^ and the region of abrupt switching was observed over less than a decade of current, whereas the PC-TFET achieves simultaneously low *I*_OFF_ and high *I*_ON_/*I*_OFF_ ratio.

### PC-TFET in gate configuration

The experimental demonstration of the PC-TFET has been achieved by fabricating and characterizing TFETs and VO_2_ switches connected as explained in the previous section (Fig. 2e,f). During the experimental tests, the gate voltage is doubly swept and the voltage of the internal node is recorded with a high impedance voltmeter in the whole range of device operation. This allows us to carefully derive the internal amplification and the effect of the MIT transition point on the TFET characteristics by extracting its intrinsic gate and drain voltages.

The TFETs used in this work are based on a strained silicon gate-all-around (GAA) nanowire (NW) technology^62, 63^ with a NW cross section of 40 × 5 nm^2^ and a gate length of 350 nm. In order to enable a low power design of the PC-TFET, it is necessary to minimize the power threshold of the MIT switch. Based on an electrothermal model considering Joule heating as the triggering mechanism for the abrupt MIT transition^26, 64^, a convenient device geometry is achieved by reducing the VO_2_ volume between the two electrodes of the MIT switch^65^. In this work such a low power actuation of a MIT switch is achieved by fabricating nanogap planar switches on a Si/SiO_2_ substrate, limiting the VO_2_ volume between the electrodes to values as low as 200 × 100 × 100 nm^3^ (see Supplementary Fig. 1 for details on the process flow and Supplementary Fig. 2 for images of a final device).

**Figure 3:**
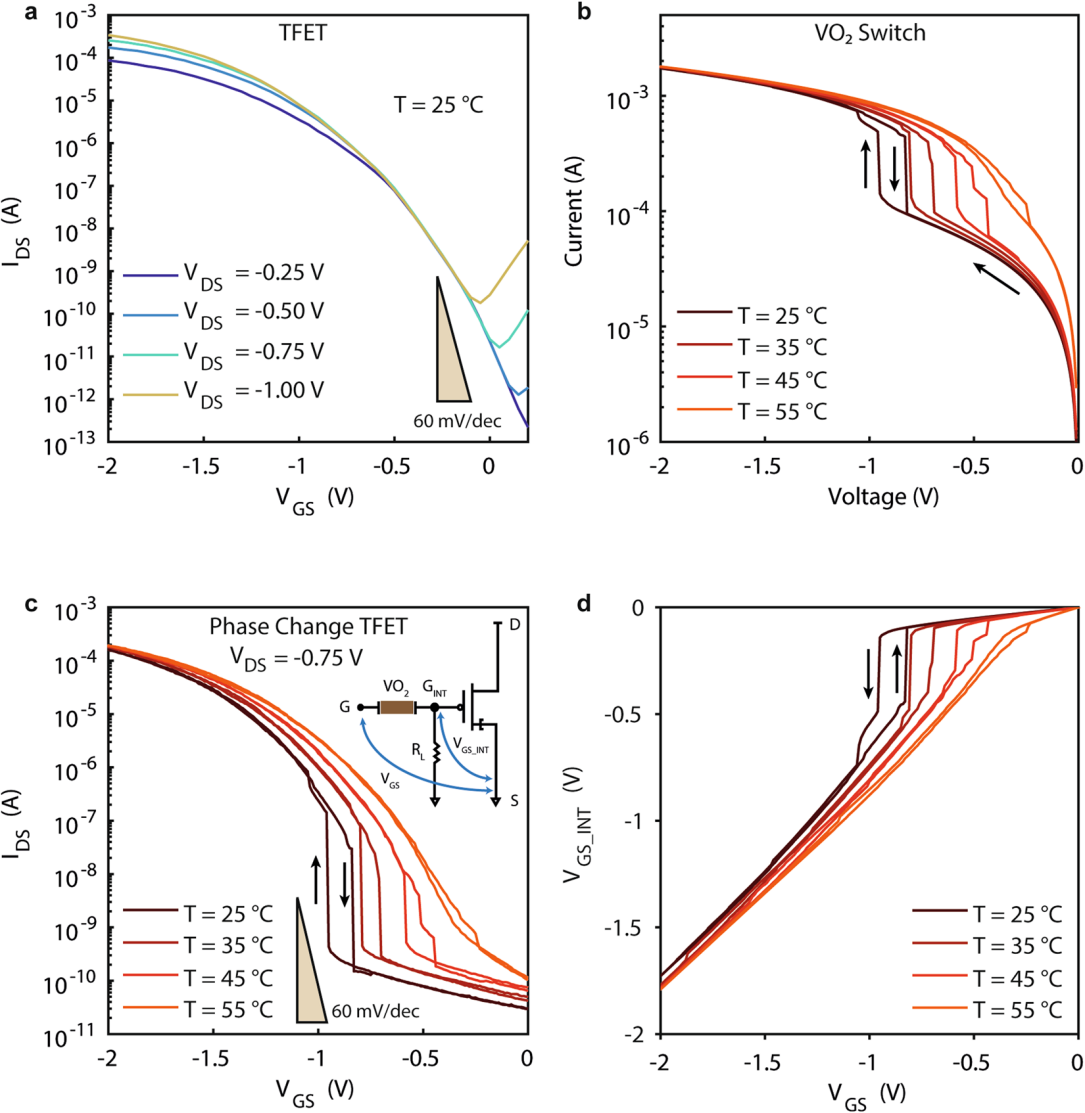
Experimental demonstration of Phase Change TFET in gate configuration. (**a**) *I*_DS_-*V*_GS_ transfer characteristic of the TFET for different applied *V*_DS_. (**b**) I-V characteristic of the VO_2_ switch measured at different temperatures with a series resistance of 1 kΩ. (**c**) *I*_DS_-*V*_GS_ obtained combining (a) and (b) in gate configuration with a load resistance of *R*_L_ = 1 kΩ between the gate terminal and ground and an applied *V*_DS_ = −0.75 V. (**d**) Internal gate voltage *V*_GS_INT_ biasing the TFET in function of the external applied *V*_GS_.

Figure 3a shows the *I*_DS_-*V*_GS_ characteristics of a TFET for different values of *V*_DS_, ranging from −0.25 V to −1 V. The TFET biased at *V*_DS_ = −0.75 V exhibits very low *I*_OFF_ = 69.1 pA, very good *I*_ON_/*I*_OFF_ = 1.0 × 10^7^ ratio, low gate leakage *I*_G_ < 8 nA up to *V*_GS_ = −2 V (see Supplementary Fig. 3), and a good average subthreshold slope over 4 decades of current: *SS*_TFET_ = ∂*V*_GS_/∂log_10_(*I*_DS_) = 112 mV/decade. Figure 3b shows the I–V characteristics of a VO_2_ switch at different temperatures, ranging from 25 °C to 55 °C. A 1 kΩ resistor is connected in series to the MIT switch in order to limit the current in the metallic state and prevent excessive overheating of the device. The switch design has been optimized for its use in the PC-TFET, presenting a low actuation voltage *V*_act_ = −0.93 V at room temperature, steep slope of the transition (*SS*_VO2_ = 18.7 mV/decade) and capability to drive high *I*_ON_ current. The transition presents limited hysteresis width (<0.2 V at room temperature) when the voltage is removed and the switch reverts to the OFF state. Increasing temperature, the actuation voltage decreases while the *I*_ON_ and the slope remain stable (*SS*_VO2_ = 17.7 mV/decade at 35 °C, 23 mV/decade at 45 °C) until reaching values near *T*_MIT_, where the sharp transition is lost. This behaviour can be explained by an electrothermal actuation model based on Joule heating^66^.

Figure 3c shows the *I*_DS_-*V*_GS_ characteristics of the PC-TFET in gate configuration at different temperatures, biased at *V*_DS_ = −0.75 V and using a load resistance *R*_L_ = 1 kΩ. Different values of *R*_L_ allow to shift the *V*_GS_act_ level necessary to induce the phase transition (as described by additional measurements reported in Supplementary Fig. 4). Once VO_2_ undergoes the phase transition to the low resistivity state, we observe a sharp rise in *I*_DS_ current up to values approaching the ones of the TFET at the same biasing conditions. The PC-TFET at room temperature has lower *I*_OFF_ = 29.5 pA (12.3 pA/µm normalized by the TFET width) than the TFET, comparable *I*_ON_/*I*_OFF_ ratio (5.5 × 10^6^) and a subthreshold slope vastly superior to the ones of state-of-the-art TFET devices reported to date: *SS*_PC_TFET_ = 4.0 mV/decade at 25 °C, 7.8 mV/decade at 45 °C. This is due to the internal amplification of *V*_GS_INT_, reported in Fig. 3d, in which we observe a very steep transition from low voltage levels to values near the TFET threshold voltage (e.g. from −0.14 V to −0.49 V at room temperature within a *V*_GS_ = 10 mV step). The output characteristics of a PC-TFET in gate configuration are reported in Supplementary Fig. 5. Due to the relatively significant power consumption in the resistive divider at the gate terminal, practically dictated by the VO_2_ actuation (see Fig. 3b), the PC-TFET in gate configuration is not providing substantial advantages for low power electronics. However, the very abrupt transition in the PC-TFET in gate configuration can be exploited for analog circuit applications such as a voltage-controlled buffered oscillator (see Supplementary Fig. 6).

### PC-TFET in source configuration

**Figure 4:**
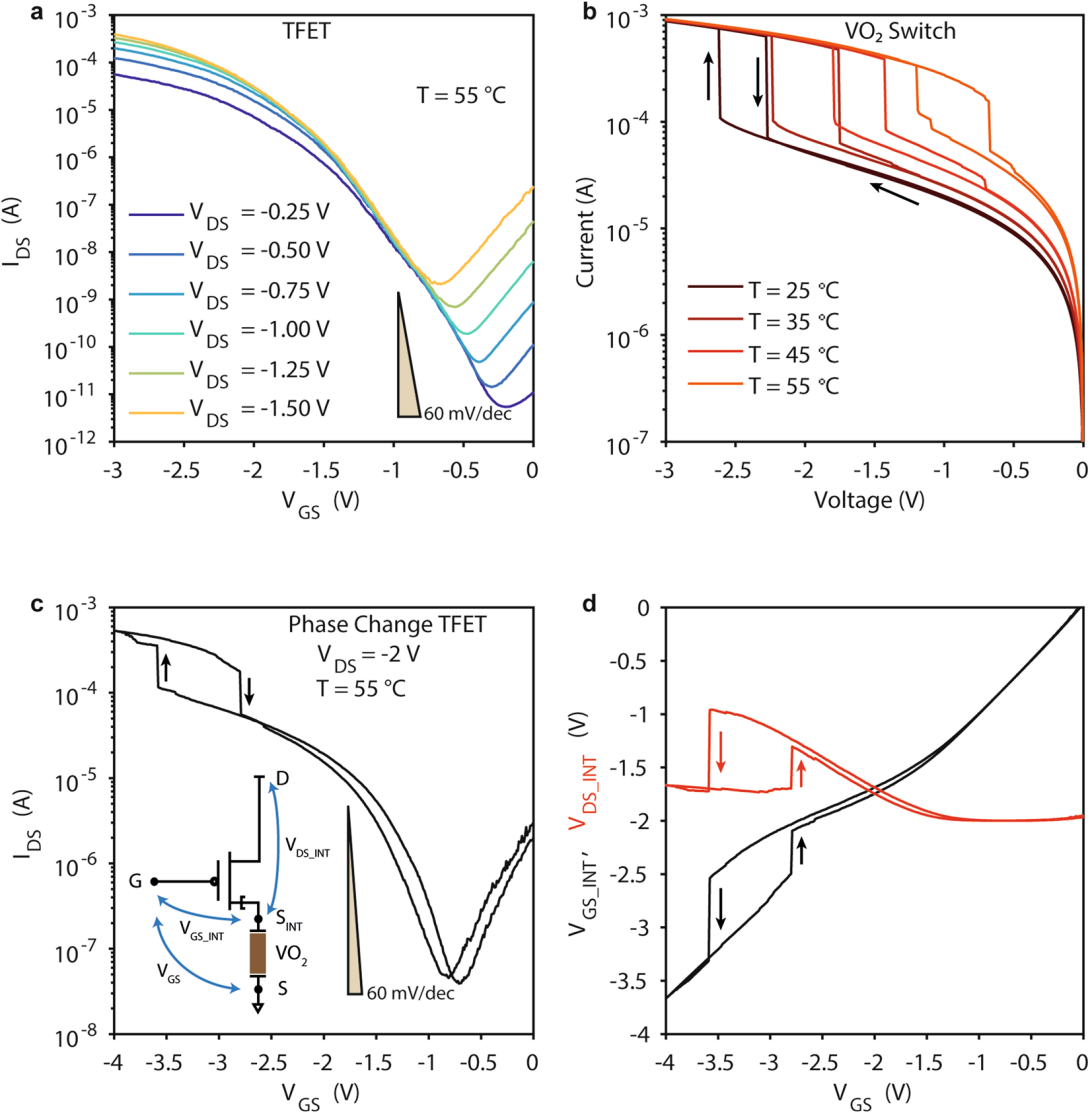
Experimental demonstration of Phase Change TFET in source configuration. (**a**) *I*_DS_-*V*_GS_ transfer characteristic of the TFET component for different applied *V*_DS_ measured at *T* = 55 °C. (**b**) I-V characteristic of the VO_2_ switch measured at different temperatures with a series resistance of 3 kΩ. (**c**) *I*_DS_-*V*_GS_ of PC-TFET obtained introducing the VO_2_ switch in the TFET source terminal; measurements performed at *T* = 55 °C with an applied external *V*_DS_ = −2 V. (**d**) Intrinsic TFET gate voltage *V*_GS_INT_ and drain voltage *V*_DS_INT_ versus applied *V*_GS_.

Figure 4a shows the *I*_DS_-*V*_GS_ characteristics of the TFET component used to implement the PC-TFET in source configuration for different values of *V*_DS_, ranging from −0.25 V to −1.5 V. The TFET measured at *T* = 55 °C and biased at *V*_DS_ = −0.75 V presents an average subthreshold swing *SS*_TFET_ ≈ 180 mV/dec and a current ratio of 6.3 × 10^5^ in a 2 V gate voltage window. Figure 4b shows the I-V characteristics of the VO_2_ switch used in this case, with a series resistance of 3 kΩ. The actuation voltage decreases with temperature from −2.61 V at 25 °C to −1.19 V at 55 °C, while the steep slope is preserved up to values approaching *T*_MIT_ (*SS*_VO2_ = 11.9 mV/decade at *T* = 25 °C, 22.3 mV/decade at *T* = 55 °C).

Figure 4c depicts the *I*_DS_-*V*_GS_ characteristics of the PC-TFET in source configuration. The *V*_DS_ has been increased to −2 V and the measurement is reported at 55 °C in order to reach the current levels necessary to induce the transition at *V*_GS_ < 4 V. The PC-TFET in source configuration combines the strengths of the two component devices, presenting a high *I*_ON_/*I*_OFF_ ratio, a low *I*_OFF_ current and a low *I*_G_ gate leakage comparable to the TFET, while the subthreshold slope is similar to the one of the VO_2_ switch (*SS*_PC_TFET_ = 20.6 mV/dec). The subthermionic (<60 mV/dec) value for the slope at the phase change transition is due to a similar internal gate voltage amplification mechanism exploited for the gate configuration, with the difference that both the intrinsic gate and drain voltages are simultaneously switching abruptly: *V*_GS_INT_ = *V*_GS_ − *R*_VO2_·*I*_D_ and *V*_DS_INT_ = *V*_DS_ − *R*_VO2_·*I*_D_ (see Fig. 4d). However, as shown in Fig. 4d, in this case the amplification occurs for values of *V*_GS_INT_ above the TFET threshold (from −2.54 V to −3.31 V within a *V*_GS_ = 10 mV step), resulting in a less abrupt increase in *I*_DS_. Moreover, our experiments show that the *V*_DS_INT_ change while sweeping *V*_GS_ is quantitatively less important than the effect of d*V*_GS_INT_/d*V*_G_ amplification (see Supplementary Fig. 7).

**Figure 5:**
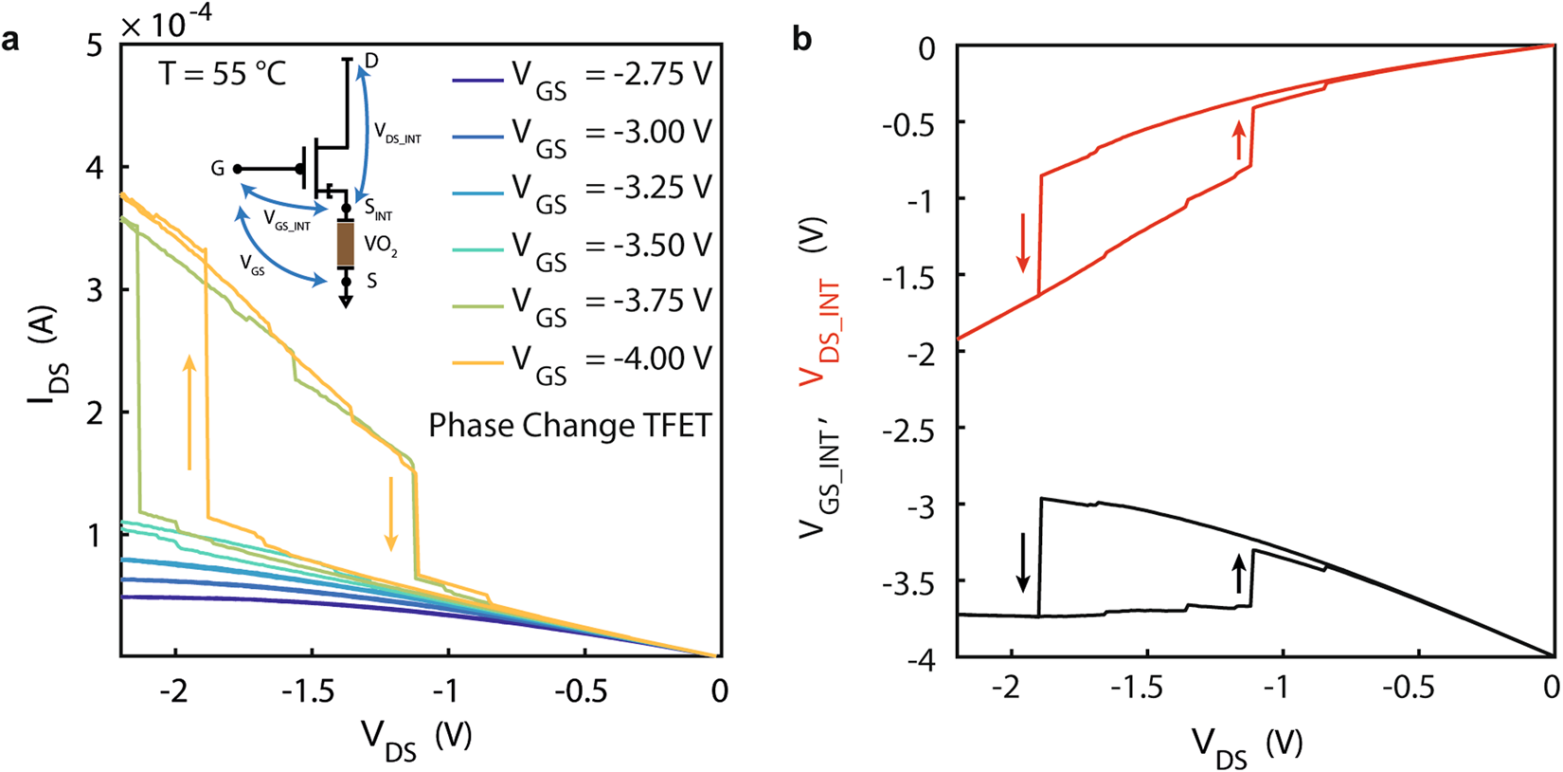
Output characteristics and drain voltage switching of Phase Change TFET in source configuration. (**a**) Output characteristics of PC-TFET in source configuration for different applied *V*_GS_ (ranging from −2.75 V to −4 V) measured at *T* = 55 °C. (**b**) Intrinsic TFET gate voltage *V*_GS_INT_ and drain voltage *V*_DS_INT_ versus applied *V*_DS_.

The output characteristics of a hybrid PC-TFET in source configuration are reported in Fig. 5a, pointing out a very particular behaviour that could be further exploited in energy efficient logic or neuromorphic circuits. The VO_2_ phase change induces a very abrupt switching in the PC-TFET output characteristics, corresponding, in absolute values, to a *higher V*_GS_INT_ and a *higher V*_DS_INT_, as pointed out by Fig. 5b. The output characteristics of PC-TFET inherit from the MIT transition points a hysteretic behaviour, which has a direct consequence on the effective drive current (because of the different trajectory on the output characteristics in logical switching) if such device is used for building CMOS inverters. Moreover, the low leakage current in the PC-TFET, negligible with respect to the drain current over the whole domain of operation (see Supplementary Fig. 8), makes it promising for energy efficient implementations of neuromorphic circuits based on relaxation oscillators^67, 68^.

### Body factor reduction in PC-TFET

**Figure 6:**
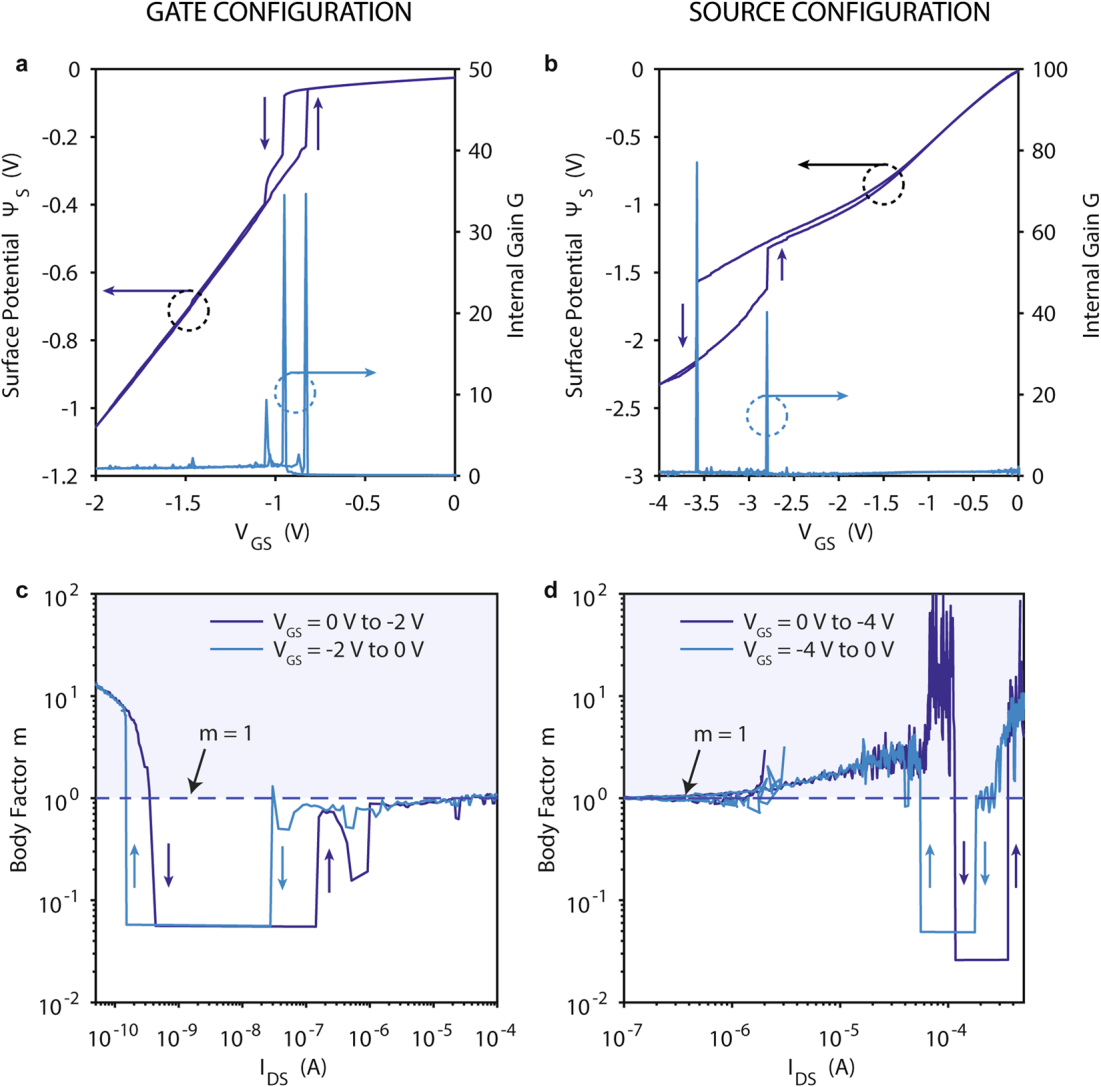
Surface potential and body factor in Phase Change TFET. (**a**,**b**) Dependence on *V*_GS_ of the surface potential, *Ψ*_S_, and internal gain, *G* = d*V*_GS_INT_/d*V*_GS_, for gate and source configurations. The reference level for the surface potential is taken at the source terminal. (**c**,**d**) Body factor as a function of *I*_DS_ for gate and source configurations; the dashed lines represent the *m* ≥ 1 limit overcome thanks to the internal *V*_GS_INT_ amplification.

The deep subthermionic switching in the PC-TFET can be explained by its sub-unity body factor due to the internal gate voltage amplification. The relation between the subthreshold slope and the body factor has been captured in equation (1), with the transistor body factor *m* = d*V*_GS_/d*Ψ*_S_ expressed as the inverse of the differential amplification of the surface potential with respect to the extrinsic gate voltage. In a conventional MOSFETs the body factor is dependent on a capacitance ratio between the gate oxide capacitance, *C*_ox_, and the depletion capacitance, *C*_d_, *m* = 1 + *C*_d_/*C*_ox_, resulting in a lower bound, *m* ≥ 1. Here, we show that this limit is overcome in the PC-TFET because the body factor *m* can be expressed in function of *V*_GS_INT_ and becomes:

2 $$m={[\frac{{\rm{d}}{\Psi }_{{\rm{S}}}}{{\rm{d}}{V}_{{\rm{GS}}\_{\rm{INT}}}}\frac{{\rm{d}}{V}_{{\rm{GS}}\_{\rm{INT}}}}{{\rm{d}}{V}_{{\rm{GS}}}}]}^{-1}=(1+\frac{{C}_{{\rm{d}}}}{{C}_{{\rm{ox}}}})\frac{d{V}_{{\rm{GS}}}}{d{V}_{{\rm{GS}}\_{\rm{INT}}}}$$

hence, when maximizing the internal gain of the PC-TFET, *G* = d*V*_GS_INT_/d*V*_GS_ >> 1, and given that in fully depleted body devices (1 + *C*_d_/*C*_ox_)~1, it follows that *m* << 1, showing that the body factor is a booster of the TFET subthreshold swing. We extract the body factor from our experimental results, starting from calculating the surface potential as a function of *V*_GS_ as shown in Fig. 6a, for the gate configuration, and Fig. 6b, for the source configuration. On the same figures we include the measured internal gain, *G*, whose experimental values are used to extract *m*, using equation (2). The values of *Ψ*_S_(*V*_GS_) are obtained by means of technology computer-aided design (TCAD) simulations of NW-TFETs identical to the fabricated structures, biased with the experimental values of *V*_GS_INT_ and *V*_DS_ for the gate configuration (Fig. 3d), *V*_GS_INT_ and *V*_DS_INT_ for the source configuration (Fig. 4d). We observe a steep change in *Ψ*_S_ (resulting in a very low *m*) in correspondence of the *V*_GS_ values for which a high internal gain amplification is recorded, highlighting the key role of the internal gain in the steep switching characteristics of the PC-TFET.

Figure 6c,d show *m* in function of the measured *I*_DS_, respectively for the gate and source configurations. In both cases the experimentally extracted body factor shows a less than 0.1 value in the transition region. The PC-TFET in gate configuration presents a value of *m* of ~0.05 (<<1) for more than two decades of current, from 0.43 nA to 142.3 nA in the OFF to ON transition and from 27.5 nA to 0.15 nA in the OFF to ON transition. The PC-TFET in source configuration shows similar values of *m* (0.025 in the OFF to ON transition, 0.5 in the ON to OFF). It is worth noting that the low-*m* region is extended for more decades of current in the gate configuration due to the better alignment of the internal gain peaks and the TFET threshold region.

## Discussion

We reported the PC-TFET as a novel hybrid steep-slope electronic switch, combining two steep switching mechanisms in a single device, and its detailed characterization in a broad range of temperatures up to values approaching the transition temperature of VO_2_. The unique combination of BTBT in TFET and MIT in VO_2_ leads to excellent figures of merit for digital electronics such as an *I*_on_/*I*_off_ ratio better than 5.5 × 10^6^ and a subthreshold swing lower than 10 mV/dec over 3 decades of currents. We observe low dependence on temperature of the swing of the PC-TFET in gate configuration, ranging from 4.0 mV/dec at room temperature to 7.8 mV/dec at 45 °C. Moreover, we have demonstrated that the underlying mechanism for the abrupt switching behaviour is the internal gate voltage amplification, leading to a sub-unity equivalent body factor. Such lower-than-1 body factor to achieve subthermionic switching is a much more general design criterion than the previous principle of negative capacitance, serving as a performance booster for both TFETs and MOSFETs. The PC-TFET represents an important step forward for beyond CMOS electronics, exploiting for the first time the full potential of the VO_2_ MIT in in an electrically gated 3-terminal architecture and opening new perspectives for low power electronics and neuromorphic computing.

## Methods

### Fabrication of experimental devices

VO_2_ nanogap switches were fabricated on a silicon substrate with a 200 nm thick SiO_2_ layer on top. The VO_2_ layer was deposited by reactive magnetron sputtering at 600 °C of a pure vanadium target, with detailed experimental conditions reported elsewhere^69^. Electrical contacts were defined by electron beam lithography on PMMA/MMA and lift-off of a 100 nm thick platinum film deposited by sputtering. The VO_2_ areas around the switch are then removed by electron beam lithography on ZEP and ion beam etching. Strained silicon GAA TFETs have been fabricated on a silicon on insulator substrate using a process based on doping segregation from NiSi_2_^70^.

### TCAD simulations for surface potential extraction

TCAD simulations were performed using Sentaurus TCAD Suite 2014.09. We simulated a strained silicon double gate TFET with channel thickness *T*_CH_ = 5 nm, oxide thickness *T*_OX_ = 3 nm with HfO_2_ (*ε*_r_ = 22) gate metal workfunction of *ϕ*_m_ = 4.1 eV corresponding to TiN. The source doping is *N*_S_ = 1 × 10^20^ cm^−3^ and the drain doping is *N*_D_ = 1 × 10^19^ cm^−3^ with abrupt junctions. Since the semiconductor layer is extremely thin, we have enlarged the bandgap by 70 meV, corresponding to the quantized state of the [100] ellipsoids. However, this increase is cancelled out by the strain on the nanowires, which results in an overall bandgap reduction of Δ*E*_g_ = −25 meV. All the simulated surface potential values reported in this work are taken from 0.1 Å below the semiconductor-oxide interface. The surface potential plots in function of *V*_GS_ (Fig. 5a,b) are taken at the tunneling junction, while the full potential profile across the channel is reported in Supplementary Fig. 9.

## Electronic supplementary material

Supplementary Information

## References

[1] Iwai, H. Technology roadmap for 22nm and beyond. In *2009 2nd Int*. *Work*. *Electron Devices Semicond*. *Technol*. 1–4, doi:10.1109/EDST.2009.5166100 (IEEE, 2009).

[2] Bernstein K, Cavin RK, Porod W, Seabaugh A, Welser J (2010). Device and Architecture Outlook for Beyond CMOS Switches. Proc. IEEE.

[3] Seabaugh AC, Zhang Q (2010). Low-voltage tunnel transistors for beyond CMOS logic. Proc. IEEE.

[4] Lundstrom, M. S. The MOSFET Revisited: Device Physics and Modeling at the Nanoscale. In *2006 IEEE international SOI Conferencee Proceedings* 1–3, doi:10.1109/SOI.2006.284404 (IEEE, 2006).

[5] Ionescu AM, Riel H (2011). Tunnel field-effect transistors as energy-efficient electronic switches. Nature.

[6] Salahuddin S, Datta S (2008). Use of negative capacitance to provide voltage amplification for low power nanoscale devices. Nano Lett..

[7] Abele, N. *et al*. Suspended-gate MOSFET: bringing new MEMS functionality into solid-state MOS transistor. In *IEEE Int*. *Electron Devices Meet*. *2005*. *IEDM Tech*. *Dig*. 479–481, doi:10.1109/IEDM.2005.1609384 (IEEE, 2005).

[8] Gopalakrishnan, K., Griffin, P. B. & Plummer, J. D. I-MOS: a novel semiconductor device with a subthreshold slope lower than kT/q. In *Dig*. *Int*. *Electron Devices Meet*. 289–292, doi:10.1109/IEDM.2002.1175835 (IEEE, 2002).

[9] Boucart K, Ionescu AM (2007). Double-gate tunnel FET with high-k gate dielectric. IEEE Trans. Electron Devices.

[10] Sarkar D (2015). A subthermionic tunnel field-effect transistor with an atomically thin channel. Nature.

[11] Newns DM (1998). Mott transition field effect transistor. Appl. Phys. Lett..

[12] Inoue IH (2005). Electrostatic carrier doping to perovskite transition-metal oxides. Semicond. Sci. Technol..

[13] Inoue IH, Rozenberg MJ (2008). Taming the mott transition for a novel mott transistor. Adv. Funct. Mater..

[14] Ha SD, Ramanathan S (2011). Adaptive oxide electronics: A review. J. Appl. Phys..

[15] Yang Z, Ko C, Ramanathan S (2011). Oxide Electronics Utilizing Ultrafast Metal-Insulator Transitions. Annu. Rev. Mater. Res..

[16] Basov DN, Averitt RD, Van Der Marel D, Dressel M, Haule K (2011). Electrodynamics of correlated electron materials. Rev. Mod. Phys..

[17] Zhou Y, Ramanathan S (2013). Correlated Electron Materials and Field Effect Transistors for Logic: A Review. Crit. Rev. Solid State Mater. Sci.

[18] Mannhart J, Haensch W (2012). Device physics: Put the pedal to the metal. Nature.

[19] Shi J, Zhou Y, Ramanathan S (2014). Colossal resistance switching and band gap modulation in a perovskite nickelate by electron doping. Nat. Commun..

[20] Morin FJ (1959). Oxides which show a metal-to-insulator transition at the neel temperature. Phys. Rev. Lett..

[21] Mott NF (1968). Metal-insulator transition. Rev. Mod. Phys..

[22] Goodenough JB (1971). The two components of the crystallographic transition in VO_2_. J. Solid State Chem..

[23] Lee SB, Kim K, Oh JS, Kahng B, Lee JS (2013). Origin of variation in switching voltages in threshold-switching phenomena of VO_2_ thin films. Appl. Phys. Lett..

[24] Simon Mun, B. *et al*. Role of joule heating effect and bulk-surface phases in voltage-driven metal-insulator transition in VO_2_ crystal. *Appl*. *Phys*. *Lett*. **103** (2013).

[25] Yoon J, Lee G, Park C, Mun BS, Ju H (2014). Investigation of length-dependent characteristics of the voltage-induced metal insulator transition in VO_2_ film devices. Appl. Phys. Lett..

[26] Jordan TS (2014). Model and Characterization of VO_2_ Thin-Film Switching Devices. IEEE Trans. Electron Devices.

[27] Rathi S (2014). Postfabrication annealing effects on insulator-metal transitions in VO_2_ thin-film devices. ACS Appl. Mater. Interfaces.

[28] Rathi S (2014). Unravelling the switching mechanisms in electric field induced insulator–metal transitions in VO_2_ nanobeams. J. Phys. D. Appl. Phys..

[29] Joushaghani A (2014). Voltage-controlled switching and thermal effects in VO_2_ nano-gap junctions. Appl. Phys. Lett..

[30] Singh S (2015). Proliferation of metallic domains caused by inhomogeneous heating near the electrically driven transition in VO_2_ nanobeams. Phys. Rev. B.

[31] Li, D. *et al*. Joule Heating-Induced Metal–Insulator Transition in Epitaxial VO_2_/TiO_2_ Devices. *ACS Appl*. *Mater*. *Interfaces* acsami.6b03501, doi:10.1021/acsami.6b03501 (2016). 10.1021/acsami.6b03501 27136956

[32] Chae BG, Kim HT, Youn DH, Kang KY (2005). Abrupt metal-insulator transition observed in VO_2_ thin films induced by a switching voltage pulse. Phys. B Condens. Matter.

[33] Leroy J (2012). High-speed metal-insulator transition in vanadium dioxide films induced by an electrical pulsed voltage over nano-gap electrodes. Appl. Phys. Lett..

[34] Zhou Y (2013). Voltage-Triggered Ultrafast Phase Transition in Vanadium Dioxide Switches. IEEE Electron Device Lett.

[35] Vitale, W. A. *et al*. Steep slope VO_2_ switches for wide-band (DC-40 GHz) reconfigurable electronics. In *72nd Device Research Conference* 29–30, doi:10.1109/DRC.2014.6872284 (IEEE, 2014).

[36] Radu IP (2015). Switching mechanism in two-terminal vanadium dioxide devices. Nanotechnology.

[37] Sakai J (2008). High-efficiency voltage oscillation in VO_2_ planer-type junctions with infinite negative differential resistance. J. Appl. Phys..

[38] Lee, Y. W. *et al*. Metal-insulator transition-induced electrical oscillation in vanadium dioxide thin film. *Appl*. *Phys*. *Lett*. **92** (2008).

[39] Shukla N (2014). Synchronized charge oscillations in correlated electron systems. Sci. Rep.

[40] Beaumont A, Leroy J, Orlianges J-C, Crunteanu A (2014). Current-induced electrical self-oscillations across out-of-plane threshold switches based on VO_2_ layers integrated in crossbars geometry. J. Appl. Phys..

[41] Bae SH (2013). The memristive properties of a single VO_2_ nanowire with switching controlled by self-heating. Adv. Mater..

[42] Seo G, Kim BJ, Kim HT, Lee YW (2014). Thermally- or optically-biased memristive switching in two-terminal VO_2_ devices. Curr. Appl. Phys..

[43] Vitale WA (2015). Steep-Slope Metal–Insulator-Transition VO_2_ Switches With Temperature-Stable High I_ON_. IEEE Electron Device Lett.

[44] Vitale, W. A. *et al*. Field-enhanced design of steep-slope VO_2_ switches for low actuation voltage. In *2016 46th European Solid-State Device Research Conference* (*ESSDERC*), 352–355, doi:10.1109/ESSDERC.2016.7599659 (IEEE, 2016).

[45] Krammer, A., Gremaud, A., Bouvard, O., Sanjines, R. & Schüler, A. *In situ* photoelectron spectroscopic characterization of reactively sputtered, doped vanadium oxide thin films. *Surf*. *Interface Anal*. 1–5, doi:10.1002/sia.5989 (2016).

[46] Kim H-T (2004). Mechanism and observation of Mott transition in VO_2_-based two- and three-terminal devices. New J. Phys..

[47] Ruzmetov D, Gopalakrishnan G, Ko C, Narayanamurti V, Ramanathan S (2010). Three-terminal field effect devices utilizing thin film vanadium oxide as the channel layer. J. Appl. Phys..

[48] Sengupta S (2011). Field-effect modulation of conductance in VO_2_ nanobeam transistors with HfO2 as the gate dielectric. Appl. Phys. Lett..

[49] Martens K (2015). Field Effect and Strongly Localized Carriers in the Metal-Insulator Transition Material VO_2_. Phys. Rev. Lett..

[50] Wei T, Kanki T, Fujiwara K, Chikanari M, Tanaka H (2016). Electric field-induced transport modulation in VO_2_ FETs with high-k oxide/organic parylene-C hybrid gate dielectric. Appl. Phys. Lett..

[51] Nakano M (2012). Collective bulk carrier delocalization driven by electrostatic surface charge accumulation. Nature.

[52] Liu K (2012). Dense electron system from gate-controlled surface metal-insulator transition. Nano Lett..

[53] Jeong J (2013). Suppression of Metal-Insulator Transition in VO_2_ by Electric Field-Induced Oxygen Vacancy Formation. Science.

[54] Karel J (2014). Distinct Electronic Structure of the Electrolyte Gate Induced Conducting Phase in Vanadium Dioxide Revealed by High Energy Photoelectron Spectroscopy. ACS Nano.

[55] Jeong J (2015). Giant reversible, facet-dependent, structural changes in a correlated-electron insulator induced by ionic liquid gating. Proc. Natl. Acad. Sci.

[56] Shibuya K, Sawa A (2016). Modulation of Metal-Insulator Transition in VO_2_ by Electrolyte Gating-Induced Protonation. Adv. Electron. Mater.

[57] Zhou Y, Ramanathan S (2012). Relaxation dynamics of ionic liquid-VO_2_ interfaces and influence in electric double-layer transistors. J. Appl. Phys..

[58] Peng X (2016). Efficient and Hysteresis-Free Field Effect Modulation of Ambipolarly Doped Vanadium Dioxide Nanowires. Phys. Rev. Appl.

[59] Casu, E. A. *et al*. Hybrid Phase-Change – Tunnel FET (PC-TFET) Switch with Subthreshold Swing <10 mV/decade and sub-0.1 body factor: digital and analog benchmarking. In 20*16 IEEE International Electron Devices Meeting* (2016).

[60] Ionescu, A. M. Ferroelectric tunnel FET switch and memory. US patent (2010).

[61] Shukla N (2015). A steep-slope transistor based on abrupt electronic phase transition. Nat. Commun..

[62] Zhao QT (2015). Strained Si and SiGe nanowire tunnel FETs for logic and analog applications. IEEE J. Electron Devices Soc.

[63] Knoll L (2014). Strained Si and SiGe tunnel-FETs and complementary tunnel-FET inverters with minimum gate lengths of 50 nm. Solid. State. Electron..

[64] Zimmers A (2013). Role of Thermal Heating on the Voltage Induced Insulator-Metal Transition in VO_2_. Phys. Rev. Lett..

[65] Joushaghani A (2015). Characteristics of the Current-Controlled Phase Transition of VO_2_ Microwires for Hybrid Optoelectronic Devices. Photonics.

[66] Vitale, W. A., Moldovan, C. F., Paone, A., Schüler, A. & Ionescu, A. M. Investigation of the Metal-Insulator Transition in VO_2_ for Electronic Switches with Sub-1 mV/Decade Steep Subthreshold Slope. In *Silicon Nanoelectronics Workshop*, doi:10.1109/SNW.2016.7578041 (2016).

[67] Shukla, N. *et al*. Pairwise coupled hybrid vanadium dioxide-MOSFET (HVFET) oscillators for non-boolean associative computing. In *2014 IEEE International Electron Devices Meeting* 28.7.1-28.7.4, doi:10.1109/IEDM.2014.7047129 (IEEE, 2014).

[68] Jerry, M. *et al*. Phase transition oxide neuron for spiking neural networks. In *74th Annual Device Research Conference* 1-2, doi:10.1109/DRC.2016.7548503 (IEEE, 2016).

[69] Vitale WA, Moldovan CF, Paone A, Schüler A, Ionescu AM (2015). Fabrication of CMOS-compatible abrupt electronic switches based on vanadium dioxide. Microelectron. Eng..

[70] Luong, G. V., Trellenkamp, S., Zhao, Q. T., Mantl, S. & Bourdelle, K. K. Strained Si nanowire GAA n-TFETs for low supply voltages. *EUROSOI-ULIS 2015 - 2015 Jt*. *Int*. *EUROSOI Work*. *Int*. *Conf*. *Ultim*. *Integr*. *Silicon* 65–68, doi:10.1109/ULIS.2015.7063774 (2015).

