# Supplementary material for: A Steep-Slope Transistor Combining Phase-Change and Band-to-Band-Tunneling to Achieve a sub-Unity Body Factor
Source: Sci Rep. 2017 Mar 23;7:355. doi: 10.1038/s41598-017-00359-6 (PMC5428222; doi:10.1038/s41598-017-00359-6)
Supplement: Supplementary file 1 — Supplementary Information [file 41598_2017_359_MOESM1_ESM.pdf]

A Steep-Slope Transistor Combining Phase-Change and Band-to-Band-Tunneling to Achieve a sub-Unity Body Factor

Wolfgang A. Vitale[1†], Emanuele A. Casu[1], Arnab Biswas[1], Teodor Rosca[1], Cem Alper[1], Anna Krammer[2], Gia V. Luong[3], Qing-T. Zhao[3], Siegfried Mantl[3], Andreas Schüler[2] & A. M. Ionescu[1]

[1] *Nanoelectronic Devices Laboratory (NanoLab), Ecole Polytechnique Fédérale de Lausanne (EPFL), 1015 Lausanne, Switzerland*
[2] *Solar Energy and Building Physics Laboratory (LESO-PB), Ecole Polytechnique Fédérale de Lausanne (EPFL), 1015 Lausanne, Switzerland*
[3] *Peter Grünberg Institut 9 (PGI-9), Forschungszentrum Jülich, 52425 Jülich, Germany*

[†] *Corresponding author; mail: wolfgang.vitale@epfl.ch*

Supplementary Figures

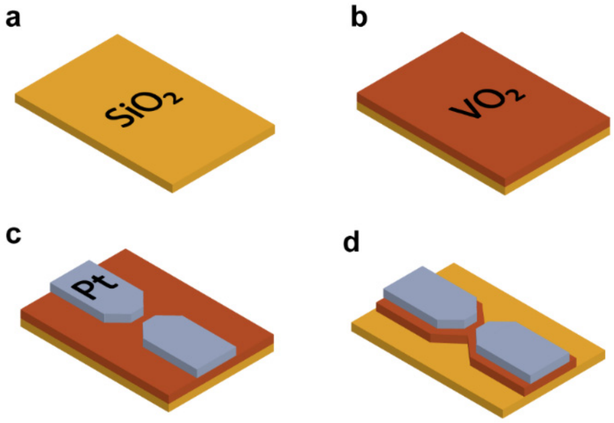

Supplementary Figure 1 | Fabrication Process of VO_2 devices. **(a)** VO_2 devices are fabricated starting from a silicon substrate with a thermal silicon oxide layer of 200 nm on top. **(b)** A 200 nm thick VO_2 layer is deposited with reactive sputtering of a Vanadium target in an O_2/Ar plasma at 600 °C substrate temperature. **(c)** Switch electrodes are defined via electron beam lithography on PMMA/MMA and lift-off of a 100 nm thick Platinum film. **(d)** VO_2 is removed around the switch area using electron beam lithography on ZEP and ion beam etching.

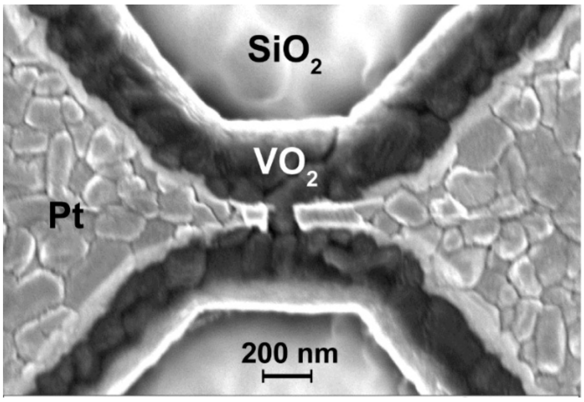

Supplementary Figure 2 | VO_2-based MIT switch. SEM image of a VO_2 switch, showing a relatively large VO_2 average grain size ~100 nm, resulting in a large and steep thermal MIT.

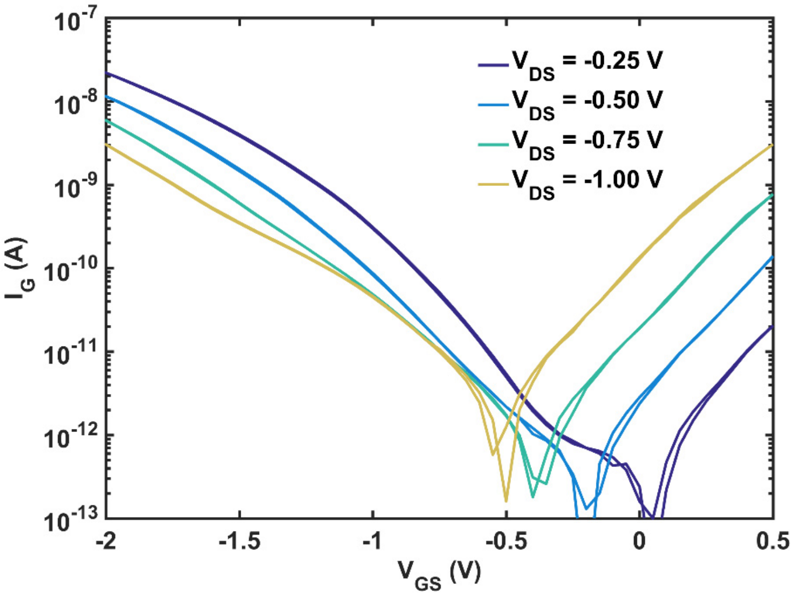

Supplementary Figure 3 | TFET gate leakage. Leakage current in the TFET used as a component for the PC-TFET for different values of drain voltage V_{DS}, ranging from -0.25 V to -1 V. Measurements performed at room temperature.

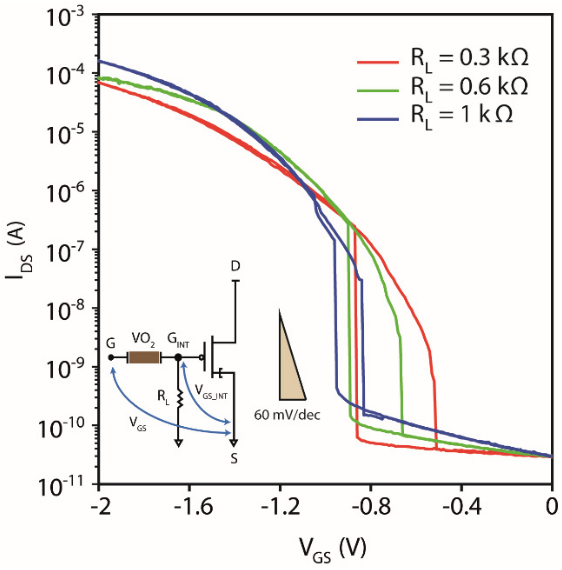

Supplementary Figure 4 | Effect of the load resistance R_L on the actuation voltage V_{GS_act} in gate configuration. I_{DS}-V_{GS} of PC-TFET in gate configuration measured at room temperature with different R_L values, ranging from 0.3 kΩ to 1 kΩ, keeping constant V_{DS} = -0.75 V.

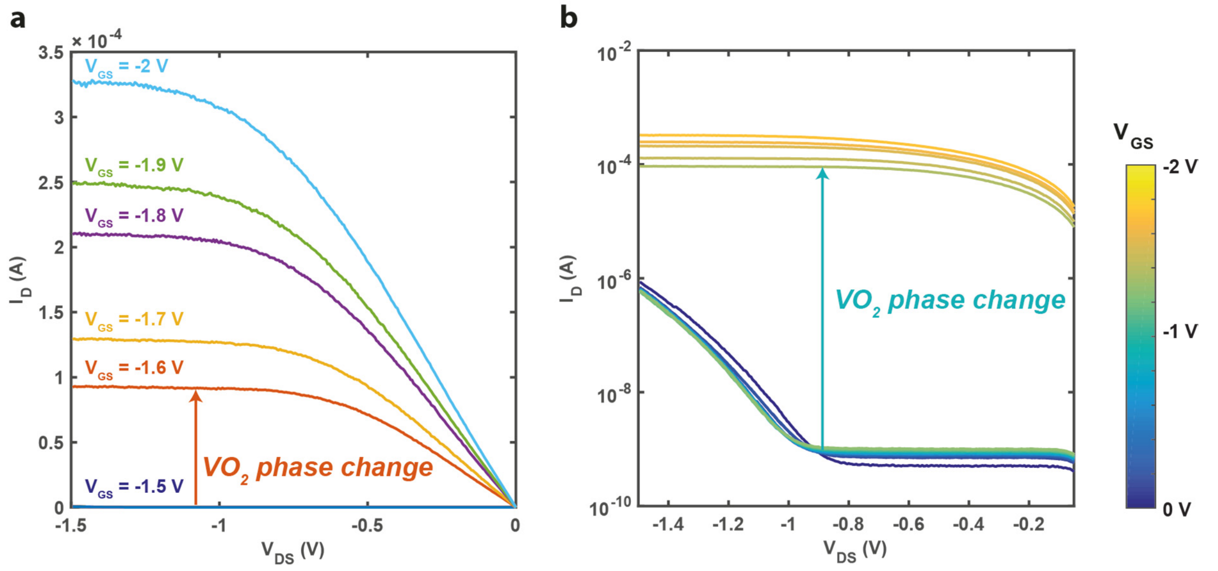

Supplementary Figure 5 | Output characteristics of PC-TFET in gate configuration. (a) Output characteristics of a PC-TFET in gate configuration for different applied V_{GS}, ranging from -0.25 V to -1 V, measured at room temperature. **(b)** Same output characteristics in logarithmic scale, to better show the effect of the phase change in VO_2.

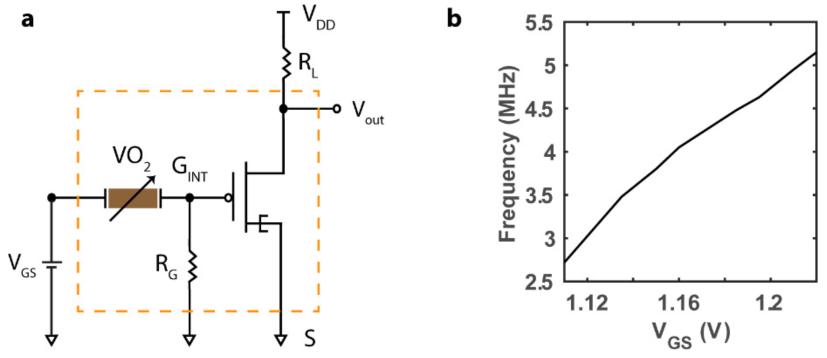

Supplementary Figure 6 | Voltage-controlled buffered oscillator based on the PC-TFET in gate configuration. (a) Circuit schematic diagram, highlighting the PC-TFET in gate configuration. The oscillation induced in the internal gate node V_{GS_INT} is read at the output V_{out}. The PC-TFET in this configuration offers a high output impedance, allowing to decouple the output load from the oscillating source. V_{GS} varied from 1.11 V to 1.22 V. R_G = 1 kΩ, R_L = 1 kΩ. R_{VO2_OFF} = 10 kΩ, R_{VO2_ON} = 100 Ω. V_{act} = 1 V, VO_2 hysteresis: 0.2 V. TFET gate capacitance C_{GS} = 50 pF. **(b)** Simulation results, showing a linear dependence of the oscillation frequency on V_{GS}, from 2.72 MHz to 5.12 MHz.

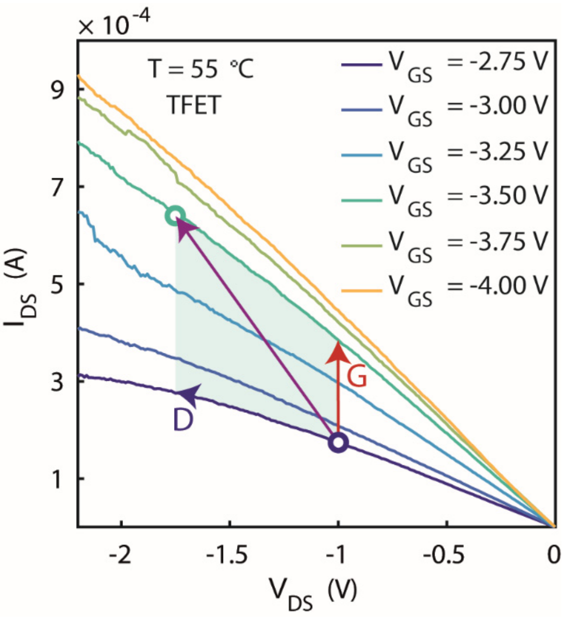

Supplementary Figure 7 | TFET output characteristics and effect of the internal voltage gains in the PC-TFET. The phase transition in VO_2 allows to switch abruptly between the two current points highlighted by a circle in the TFET output characteristics. This abrupt current increase is due to the combined effect of the gate voltage gain $G = dV_{GS\_int}/dV_{GS} = 75$ (red arrow) and the drain voltage gain $D = dV_{DS\_int}/dV_{GS} = 75$ (blue arrow). The values of G and D correspond to the ones measured in the PC-TFET in source configuration. Even if $G = D$, the gate voltage gain has a higher effect on the current increase.

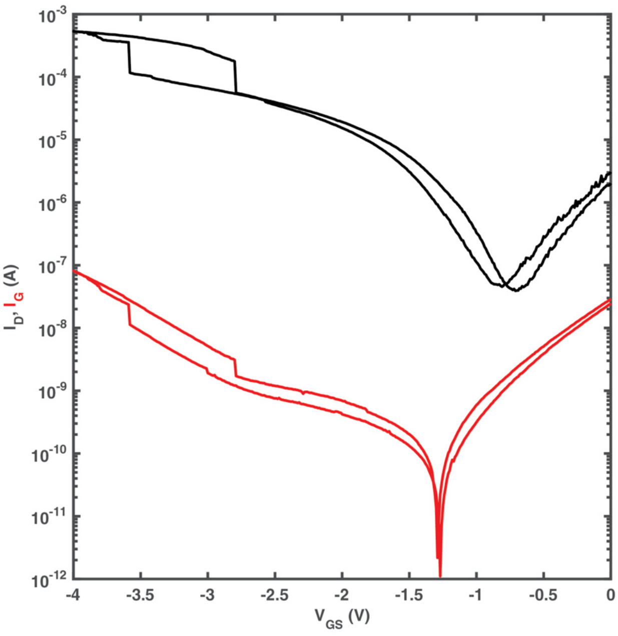

Supplementary Figure 8 | Gate leakage for the PC-TFET in source configuration, compared to drain current. Gate leakage (red) and drain current (black) in function of V_{GS} for the PC-TFET in source configuration. Measurements performed at T = 55 °C with an applied external V_{DS} = -2 V. The gate leakage is negligible compared to the drain current over the whole domain of operation of the PC-TFET.

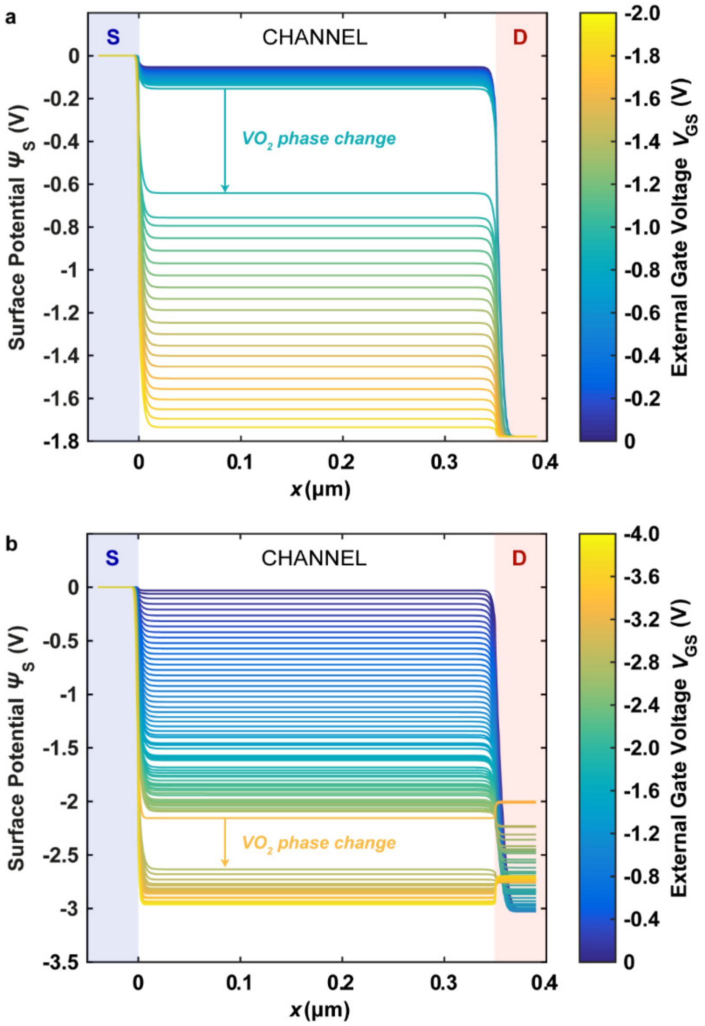

Supplementary Figure 9 | Potential cuts along the TFET channel. (a) Gate configuration. External gate voltage V_{GS} varying from 0 V to -2 V. External drain voltage V_{DS} = -0.75 V. Measurements performed at T = 25 °C. **(b)** Source configuration. External gate voltage V_{GS} varying from 0 V to -4 V. External drain voltage V_{DS} = -2 V. Measurements performed at T = 55 °C.
